# Viremia copy-years and risk of estimated glomerular filtration rate reduction in adults living with perinatal HIV infection

**DOI:** 10.1371/journal.pone.0240550

**Published:** 2020-10-15

**Authors:** Giovanni Sarteschi, Antonio Di Biagio, Emanuele Focà, Lucia Taramasso, Francesca Bovis, Anna Celotti, Michele Mirabella, Laura Magnasco, Sara Mora, Mauro Giacomini, Matteo Bassetti

**Affiliations:** 1 Department of Health Sciences (DISSAL), University of Genoa, Genoa, Italy; 2 Infectious and Tropical Diseases Department, University of Brescia and ASST Spedali Civili Hospital, Brescia, Italy; 3 Department of Pathophysiology and Transplantation, Infectious Diseases Unit, Fondazione IRCCS Ca’ Granda Ospedale Maggiore Policlinico, University of Milan, Milan, Italy; 4 MultidisciplinAry ResearCh in Health Science (MACH), Milan, Italy; 5 Biostatistics Unit, Department of Health Sciences, University of Genoa, Genoa, Italy; 6 Department of Informatics Bioengineering, Robotics and System Engineering (DIBRIS), University of Genoa, Genoa, Italy; 7 Infectious Diseases Unit, IRCCS Ospedale Policlinico San Martino Hospital, Genoa, Italy; Boston University, UNITED STATES

## Abstract

Among people with perinatal HIV infection (PHIV), non-communicable diseases, such as chronic kidney disease, are increasing. Both HIV replication and antiretroviral therapy are recognised causes of renal impairment. Objective of the study is to describe the impact of viremia copy-years (VCY) and antiretroviral therapy on trend of estimated glomerular filtration rate (eGFR) in a cohort of adults with perinatal HIV infection. We conducted a multicentre observational study in sixty adults living with PHIV across a 9-year period, from January 2010 to December 2018. The mean values of eGFR were analysed at the first (T0) and last year of observation (T1). VCY was defined as the area under HIV-RNA curve during the study period. We analysed data according to antiretroviral therapy: tenofovir disoproxil (TDF), non-nucleoside reverse transcriptase inhibitors (NNRTI), boosted protease inhibitors (PI/b), integrase inhibitors (INI). We observed a mean overall eGFR reduction from 126.6 mL/min (95%CI: 119.6–133.5) to 105.0 mL/min (95%CI: 99.55–110.6) (*p*<0.001). Older age, higher baseline eGFR, higher VCY and longer exposure to INI treatment were associated with eGFR reduction at univariate analysis. In the multivariate model, older age (*p* = 0.039), baseline eGFR (*p*<0.001) and VCY (*p* = 0.069), were retained. We also observed a longer exposure to PI/b and INI in patients with lower control on HIV-RNA, expressed as VCY>2 log_10_. Our study outlines a progressive eGFR reduction in young adults with PHIV, related to the lower control on HIV-RNA VCY and related to aging.

## Introduction

Since the early 2000s, there has been a gradual reduction of perinatal HIV infection (PHIV), owing to the extensive use of modern antiretroviral therapy (ART) in pregnant women and prophylaxis in newborns [1, 2]. Parallel to the declining prevalence of perinatal HIV infection (PHIV) in the youngest age group, there has been an increase in the number of adolescents and young adults living with HIV, whose better quality of life and longer survival periods can be attributed to the immune-virological success of modern ART [3, 4]. Currently, the prevention of premature aging and long-term adverse outcomes is becoming increasingly important. One of these outcomes is chronic kidney disease (CKD), usually defined as a pathological decline in estimated glomerular filtration rate (eGFR) [5–7]. The normal eGFR in young adults is approximately 125 mL/min and an age-related eGFR decline of 1.49±0.61 mL/min per decade is described in the literature [8, 9]. Another key objective in patients with PHIV is maintaining the HIV-RNA below 50 copies/mL, which is often challenging due to the high frequency of drug resistance mutations since it is well established that a higher viremia copy-years (VCY)–expression of the cumulative HIV-RNA load over time—is associated with increased morbidity and mortality [10–13]. Tenofovir disoproxil fumarate (TDF) is widely prescribed for patients with PHIV and usually associated in ART with non-nucleoside reverse transcriptase inhibitors (NNRTI), boosted protease inhibitors (PI/b) or integrase inhibitors (INI). The HIV infection itself is a recognized cause of renal disease, as well as TDF and PI/b use, especially when combined in the same ART regimen [14–18]. Several ART regimens inhibit tubular creatinine secretion, resulting in a stable mild reduction in eGFR without other manifestations of renal damage [19, 20]. However, little is known about the correlation between TDF use and the trend of estimated glomerular filtration rate (eGFR) in patients with PHIV. Moreover, data are lacking on renal function in young adults growing up with PHIV and years of chronic exposure to ART, in general, and to TDF, in particular [21–23]. Our study seeks to describe the impact of VCY in the eGFR trend in a multicentric cohort of adults living with PHIV across a 9 years’ observation period and to investigate the possible influence of different ART strategies (TDF-, NNRTI-, INI- or PI/b-containing regimens) on this trend.

## Materials and methods

This is an observational, retrospective, multicentric study performed over a 9-year period, from January 2010 to December 2018. We enrolled patients with PHIV in follow up for at least 4 years in two different Italian Infectious Diseases centres: Policlinico San Martino Hospital in Genoa and ASST Spedali Civili Hospital in Brescia. The MedInfo online platform (www.reteligureHIV.it)–an online database for anonymous and automatic data collection—was used to retrieve clinical data in Genoa [24]; in Brescia the electronic medical record NetCare was used. We analysed the mean eGFR values of the first and last year data available for each patient from 2010 to 2018, defining them as T0 and T1. We calculated eGFR with the Cockcroft Gault equation in patients >18 years in 2018; for the only patient underage for the entire study, we used the revised Schwartz equation [25, 26]. CKD was defined as a value of eGFR <60 mL/min for >3 months [8]. In order to consider all the possible fluctuations in viral load over the long observation time, we chose to express the cumulative viral load per patient as VCY. VCY is a dynamic indicator defined as the area under the longitudinal HIV-RNA (copies/mL) curve during the study period (2010–2018) and expressed as log_10_ [10, 11]. We collected all HIV-RNA values available in the period analysed (2010–2018) for each patient. All the values of HIV-RNA ranging between 1–49 copies/mL were considered non-detectable and thus transformed to 1 in the computation of VCY (log_10_ = 0). We also collected data on risk factors for renal impairment (previous notification of acquired immunodeficiency syndrome [AIDS]-defining illnesses irrespective of CD4^+^T-lymphocytes count, smoking, drug abuse, hepatitis C virus [HCV] co-infection, hypertension, diabetes, known renal disease), and ART (TDF, NNRTI, INI or PI/b use). Patients were dived into two subgroups based on log_10_VCY using 2 –the median value of VCY in the participants with PHIV—as the cut-off. Potential predictors of eGFR change were selected at the univariable analysis and predictors with a p-value <0.10 were included in the multivariable model. The study was performed in accordance with the ethical standards laid down in the 1964 Declaration of Helsinki and its later amendments and in accordance with Italian national laws. All patients studied in both hospitals signed an informed consent form in which they agreed to use of their clinical data in an anonymous form for scientific purposes. The use of the Ligurian HIV Network database for scientific purposes was approved by the Ligurian Ethics Committee (date of approval: 28 August 2013). For underage patients, parents or legal guardians provided their consent for the processing of medical records.

## Results

Among the 72 patients with PHIV followed during the study period, 12 were excluded because of missing data and 60 were considered for the study. The mean time of observation was 8 years (range 4–9). Patients’ characteristics are outlined in Table 1. In our cohort, 35 were female (58%) with a mean age at baseline of 18.9 years (±4.4, range 6–28); European origin was the most represented (88%, n = 53). Eleven of our female patients (31%) had an history of pregnancies, more than one in seven cases (12%). Mean nadir of CD4^+^ T-lymphocytes (CD4^+^) was 330 (±188) cells/mm^3^, 13 patients (22%) had a CD4+ nadir <200 cells/mm^3^ and 12 (20%) had a previous diagnosis of AIDS-defining illness (3 HIV dementia complex, 3 progressive multifocal leukoencephalopathy, 1 atypical disseminated mycobacteriosis, 1 *Pneumocystis jirovecii* pneumonia, 1 recurrent bacterial pneumonia, 1 disseminated cryptococcosis, 1 extrapulmonary tuberculosis, 1 neurotoxoplasmosis). The median VCY (log_10_) was 2.0 (IQR 1.2 to 3.6), while 58 patients (96.7%) had HIV-RNA <50 copies/mL in 2018. Overall, across 9 years of study, we observed a significant decrease in the eGFR mean values from 126.6 mL/min (95%CI: 119.6–133.5) to 105.0 mL/min (95%CI: 99.55–110.6) (p < .0001, the model was adjusted for the length of the follow-up and age at baseline). An inverse correlation between eGFR trend and VCY was observed, with a lesser eGFR reduction in patients with lower VCY value (log_10_ ≤2), median -12.7 mL/min (IQR -31.0 to 2.3), compared to those with higher VCY value (log_10_ >2), -25.4 mL/min (IQR -38.3 to -14.5), (*p* = 0.027, the model was adjusted for the length of the follow-up). Patients with VCY >2 log_10_ had major median exposure to PI/b (7 years, IQR 5 to 9) and INI (4 years, IQR 3 to 7) and lower to TDF (4 years, IQR 3 to 6) and NNRTI (1 years, IQR 0 to 4) compared to those with VCY ≤2 log_10_ (respectively, a median exposure to TDF of 8 years [IQR 3 to 8], to NNRTI of 5 years [IQR 0 to 9] and of 0 years to PI/b [IQR 0 to 8] and INI [IQR 0 to 0]). At the univariate model, the following 4 predictors exhibited consistent association with eGFR change over time: major age at baseline (beta coefficients of -1.83 [95%CI -3.29; -0.37, *p* = 0.015]), a greater eGFR at baseline (-0.51 [95%CI -0.67; -0.34, *p*<0.001]) a greater VCY (-3.90, [95%CI -7.75; -0.04, *p* = 0.048]) and greater years of treatment with INI (-1.93 [95%CI -4.09; -0.23, *p* = 0.079])(Table 2). In the multivariable model, the years of INI were not retained, while a greater age (-1.21 [95%CI: -3.02; -0.10], p = 0.037), eGFR at baseline (-0.46 [95%CI: -0.62;-0.30], p < .0001) and VCY (-2.75 [95%CI: -5.73; 0.22], p = 0.069) indicated association with eGFR decline. No patients developed CKD (eGFR<60 mL/min for >3 months) during the study period, while eGFR worsened in 13 (22%) to <90 mL/min.

**Table 1:** Patients’ characteristics.

FEATURE	TOTAL n = 60 (100%)
**Age**	
Mean age at baseline (years)	18.9 [SD 4.4]
Underage at 2010	14 (23)
**Sex**	
Male	25 (42)
Female	35 (58)
**Race**	
European	53 (88)
Non-European	7 (12)
African	4 (7)
Latin	3 (5)
**Renal damage risk factors**	
Smoke	20 (33)
Drug abuse	5 (8)
**Comorbidities**	
Hypertension	2 (3)
HCV co-infection	6 (10)
Renal disease^a^	1 (2)
Diabetes	0 (0)
**Immunological status**	
Mean CD4^+^ nadir	330 cells/mm^3^ [SD 188]
CD4^+^ nadir <200	13 (22)
CD4^+^ nadir >200	47 (78)
Clinical AIDS diagnosis^b^	12 (20)
**Virological status**	
Median VCY	2.0 log_10_ [IQR 1.2–3.6]
VCY≤2 log_10_	30 (50)
VCY>2 log_10_	30 (50)
HIV-RNA<50 cp/mL at 2018	58 (97%)

AIDS = acquired immunodeficiency syndrome; CD4^+^ = CD4^+^ T-lymphocytes; VCY = copy-years viremia; HCV = hepatitis C virus; IQR = inter-quartile range; SD = standard deviation.

^a^ the only patient with renal disease included in this study had a lupus nephritis.

^b^ Causative agents for AIDS notification: 3 HIV dementia complex, 3 progressive multifocal leukoencephalopathy, 1 atypical disseminated mycobacteriosis, 1 Pneumocystis jirovecii pneumonia, 1 recurrent bacterial pneumonia, 1 disseminated cryptococcosis, 1 extrapulmonary tuberculosis, 1 neurotoxoplasmosis.

**Table 2:** Potential predictors of eGFR reduction in PHIV patients.

	Univariate analysis		Multivariate analysis	
	Beta coefficients (95% CI)	*p*-value	Beta coefficients (95% CI)	p-value
**Age at baseline**	-1.83 (-3.29; -0.37)	**0.015**	-1.21 (-3.02; -0.10)	0.037
**eGFR baseline**	-0.51 (-0.67; -0.34)	**<.0001**	-0.46 (-0.62; -0.30)	<.0001
**Male sex**	10.53 (-2.90; 23.96)	0.122		
**Current smoking**	0.10 (-14.24; 14.44)	0.989		
**Drug abuse**	8.81 (-15.54; 33.15)	0.472		
**Presence of comorbidity**	-9.29 (-28.06; 9.47)	0.326		
**Clinical AIDS diagnosis**	-4.84 (-21.68; 12.01)	0.568		
**Copy-years (log-transformed)**	-3.90 (-7.75; -0.04)	**0.048**	-2.75 (-5.73; 0.22)	0.069
**CD4**^**+**^ **nadir**	0.02 (-0.02; 0.05)	0.326		
**Years of TDF therapy**	0.38 (-1.91; 2.67)	0.738		
**Years of TDF+PI/b therapy**	-0.57 (-3.46; 2.31)	0.692		
**Years of PI/b therapy**	-0.86 (-2.74; 1.03)	0.366		
**Years of NNRTI therapy**	0.08 (-1.79; 1.94)	0.933		
**Years of INI therapy**	-1.93 (-4.09; -0.23)	**0.079**		
**Never TDF**	-6.77 (-29.22; 15.69)	0.549		

AIDS = acquired immunodeficiency syndrome; INI = integrase inhibitors; eGFR = estimated glomerular filtration rate; NNRTI = non-nucleoside reverse transcriptase inhibitors; PHIV: perinatal HIV infection; PI/b = boosted protease inhibitors; TDF = tenofovir disoproxil fumarate.

## Discussion

In this analysis, we offer new data about cumulative VCY and eGFR trend among people with PHIV. Our analysis is one of the first describing eGFR trend in adult patients with PHIV. This trend is expected to be worse compared to that of a healthy population, where factors of kidney damage such as ART exposure and HIV infection are absent [8, 9]. In previous studies on adolescents and young adults living with PHIV, TDF was associated with an eGFR decline similar to that found in our patients (approximately 1–6 mL/min/year). However, the possible role of VCY and other antiretrovirals associated with TDF had not been adequately studied yet [21–23]. In our study, a significant worsening in renal function was observed in all participants, irrespective of the type of prescribed ART, while no patients developed CKD (eGFR<60 mL/min for >3 months) during the study period (the only patient with CKD had eGFR<60 mL/min at baseline). As expected, patients with lower VCY, and hence better control of HIV infection over time, reported a lesser deterioration in renal function compared to patients with VCY ≥2 log_10_. Patients with higher VCY values had longer PI/b and INI exposure. This can perhaps be attributed to the higher frequency of resistance mutations in viral genotype, common among patients with PHIV exposed to older, suboptimal ART regimens for decades [11]. Furthermore, clinicians generally prefer a PI/b- or INI- or both- based regimen (with higher genetic barrier) in less adherent patients. In the univariate analysis, we identified a relation between years of treatment with INI and eGFR reduction, while it was not observed in other ART regimens. Notably, both elvitegravir/cobicistat and dolutegravir have been associated with increased creatinine levels—though without a worsening of renal function—due to the inhibition of creatinine tubular secretion [20, 27–29]. However, as shown in the multivariate analysis, higher VCY—and not years of exposure to INI—was retained as the predictor of eGFR reduction, thereby confirming the significant role of HIV replication in eGFR decline in the PHIV population studied. Indeed, most patients were virologically suppressed (HIV-RNA<50 copies/mL) at T1; however, a great proportion (50%) showed a history of viral blips or virological failures, expressed by VCY value >2 log_10_. Data on the effect of VCY on eGFR trend are lacking in the literature. However, a greater VCY has been correlated with hypertension and higher mortality [10, 11]. We suggest that HIV replication, also in the case of low-level viremia, correlates with greater eGFR reduction, as suggested by the tendency showed in the multivariate analysis (*p* = 0.069). A previous clinical diagnosis of AIDS, a lower CD4+ nadir, drug abuse or current smoking were not related, in our analysis, to eGFR reduction. Our data suggests that monitoring renal function in people with PHIV is mandatory despite the ART regimen prescribed, and that maintaining viral suppression could be a determinant factor in preserving renal function in patients with PHIV. Limitations of this study are its retrospective nature that restricts us from evaluating the influence of possible unmeasured confounders on the eGFR trend, the absence of a control group that prevents us from comparing the worsening trend observed among the studied PHIV patients with a healthy population and the absence of other laboratory markers of renal damage.

Our study outlines a progressive eGFR reduction among young adult patients with perinatal HIV infection, related to higher VCY and to aging. More efforts are needed in monitoring renal function and maintain stable viral suppression in patients with PHIV.

## Abbreviations

AIDS: acquired immunodeficiency syndrome
ART: antiretroviral therapy
CD4^+^: CD4^+^ T-lymphocytes
CKD: chronic kidney disease
eGFR: estimated glomerular filtration rate
HCV: hepatitis C virus
HIV: human immunodeficiency virus
INI: integrase inhibitors
NNRTI: non-nucleoside reverse transcriptase inhibitors
NRTI: nucleotide reverse transcriptase inhibitors
PHIV: perinatal HIV infection
PI/b: boosted protease inhibitors
TDF: tenofovir disoproxil fumarate
VCY: viremia copy-years
